# Extended Inter-Meal Interval Negatively Impacted the Glycemic and Insulinemic Responses after Both Lunch and Dinner in Healthy Subjects

**DOI:** 10.3390/nu14173617

**Published:** 2022-09-01

**Authors:** Xuejiao Lu, Zhihong Fan, Anshu Liu, Rui Liu, Xinling Lou, Jiahui Hu

**Affiliations:** 1 College of Food Science and Nutritional Engineering, China Agricultural University, Beijing 100083, China; 2 Key Laboratory of Precision Nutrition and Food Quality, Department of Nutrition and Health, China Agricultural University, Beijing 100083, China

**Keywords:** meal timing, inter-meal interval, apple preload, glucose, insulin

## Abstract

This study aimed to investigate the glycemic and insulinemic effects of lunch timing based on a fixed feeding window, and the effects of apple preload on postprandial glucose and insulin responses after nutrient-balanced lunch and the subsequent high-fat dinner in healthy participants. Twenty-six participants completed four randomized, crossover experimental trials: (1) early standardized lunch at 12:00 (12S); (2) apple preload to 12S (12A+S); (3) late standardized lunch at 14:00 (14S); and (4) apple preload to 14S (14A+S); wherein twenty participants’ blood samples were collected for insulin analysis following the lunch trails. In each experimental trial, each participant equipped with a continuous glucose monitor (CGM) was provided with a standardized breakfast and a high-fat dinner to be consumed at 8:00 and 18:00, respectively. The late lunch (14S) resulted in significantly elevated glucose peak, delayed insulin peak time, decreased insulin sensitivity, and increased insulin resistance following the lunch; also decreased glycemic response following the subsequent dinner and larger blood glucose fluctuation over the 24-h period compared with the 12S. The 14A+S significantly reduced the glucose peak, the insulin peak time and the glycemic variability following the lunch, also the 24-h glycemic variability compared with the 14S. The insulin sensitivity was significantly improved in the 12A+S, compared with that of the 12S. In conclusion, the present study found that an extra 2-h inter-meal fasting before and after lunch resulted in elevated glycemic response in both macronutrient-balanced meal and high-fat meal in healthy subjects. The negative impact of a late lunch could be partly reversed by the apple preload, without a trade-off of insulin secretion.

## 1. Introduction

Obesity and hyperglycemia are regarded as among the most concerning public health issues in most parts of the world [1,2]. Recently, the timing of food ingestion, in addition to the composition of food, has been proposed as a crucial determinant of the postprandial metabolic parameters associated with glycemic control and body weight management [3,4].

Evidence from epidemiological studies is accumulating that the late eating is associated with elevated risk of obesity, diabetes and cardiovascular diseases [5,6,7,8]. Several trials indicated that the glycemic homeostasis and insulin sensitivity tend to deteriorate in the later part of the day in healthy subjects [9,10,11]. Low glycemic meal could elicit greater glucose response and insulin response when consumed at 22:00 than it could when consumed at 18:00 [12]. Previous studies suggested that early dinner would be beneficial to keep a mild postprandial glycemic response and a stable blood glucose level at night [13,14], while a shortened feeding window in time-restriction feeding would help to lose weight and improve glycemic control [15].

With respect to the effect of the meal timing on metabolic consequences, most trials focused on the dinner time or the feeding window between breakfast and dinner [3,16]. It is well accepted that the timing of dinner plays a significant role in blood glucose regulation. However, the timing of lunch, which sets the inter-meal fasting period after breakfast and before dinner, was yet to be fully explored. Limited studies referring the postprandial glycemic response of lunch gave inconsistent results regarding the postprandial glycemic response after lunch and the subsequent meal [9,17,18]. Given that the time setting of the meals were different among studies, it is not easy to precisely compare the results in different studies, as the confounders of unequal meal intervals and feeding windows cannot be ruled out.

Several epidemiological studies have suggested that late lunch patterns were related to less effective weight-loss [19,20,21], increased insulin resistance [19], and the risk of polycystic ovary syndrome [22]. A few randomized controlled trials investigating different lunch timing demonstrated that late lunch resulted in decreased glucose tolerance, disturbed stress hormone rhythm [23], negative body composition change, and reduced microbiota diversity in healthy women [24]. However, there is paucity of data regarding the impact of the inter-meal interval on the postprandial glucose and insulin response and the glycemic excursion over the 24-h period, based on the same feeding window in real life settings.

It has been already proved that preload can be a convenient dietary strategy to improve postprandial glucose homeostasis [25,26]. Previous studies reported that the fruits and dried fruits such as apple [27,28,29], when consumed 30 min prior to a rice meal, significantly mitigated the postprandial glycemic response in extents of 31.4% to 50% cut of the incremental area under the curve (iAUC). However, it had not yet been confirmed whether the fruit preload could exert a remarkable glycemic stabilizing effect in nutrient-balanced mixed meals with different mealtime settings.

In the present study, we set the early lunch at 12:00 and the late lunch at 14:00, with a fixed feeding window of 10 h (8:00 to 18:00). The glycemic and insulinemic effects of lunch timing, and the effects of the apple preload on postprandial glucose and insulin responses after nutrient-balanced lunch and the subsequent high-fat dinner, were investigated in healthy participants. We supposed that the inter-meal interval would pose an impact on postprandial glycemic response based on a fixed feeding window. The first research hypothesis was that an extended before-meal fasting might resulted in exaggerated postprandial blood glucose excursion in the context of lunch and dinner, irrespective of the macronutrient composition of the meal. The second hypothesis was that the apple preload would partly reverse the negative glycemic and insulinemic responses elicited by a prolonged inter-meal fasting.

## 2. Materials and Methods

### 2.1. Participants and Ethics

Participants of both sexes volunteered via social media advertisements and were included if the following criteria were satisfied: generally healthy university students with a normal body mass index (BMI) between 18.5 and 24.0 kg/m^2^ [30]; a regular sleep–wake cycle; consuming 3 meals a day with lunch no later than 13:00 and dinner no later than 19:00; and a regular menstrual cycle (if female). The following exclusion criteria were applied to select the potential participants: allergies or intolerance to any of the test foods; a change in body weight of more than 5 kg within the past six months; any metabolic diseases covering diabetes, hypertension, metabolic syndrome; gastrointestinal disorders such as gastroesophageal reflux disease; heavy drinking; active smoking; use of medications or supplements known to affect circadian rhythms or metabolism; sleeping and eating disorders; engagement in competitive or endurance sports.

All participants interested in the study completed a questionnaire on daily lifestyle habits (dietary, sleep, physical activity) and health status by a face-to-face interview for an initial pre-screening. After that, eligible participants attended the lab for a morning, fasted, baseline tested visit prior to the first trial condition where a 2-h oral glucose tolerance test (OGTT) was administered. Before the test, body mass and fat mass were measured using a body fat scale (HBF-371, OMRON, Yangzhou, China) where the visceral fat index and basal metabolic rate (BMR) were obtained in the meantime. Resting blood pressure was measured in duplicate using an electronic blood pressure monitor (HEM-7200, OMRON, Dalian, China). Waist and hip circumferences were measured using a tape measure in duplicate to the nearest 0.1 cm.

The study protocol was conducted according to the principles laid down in the Helsinki Declaration, granted by the Ethics Committee of China Agricultural University (ethics number CAUHR-20220202), and registered at the Chinese Clinical Trial Registry (ChiCTR2200057791). All eligible individuals provided the written informed consent.

### 2.2. Study Design and Procedures

**Figure 1:**
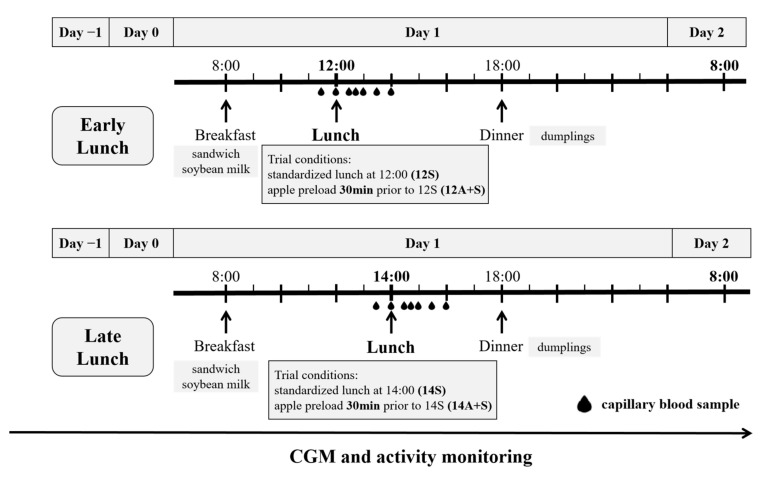
Summary of the study experimental design. CGM, continuous glucose monitor. All participants underwent four lunch trial conditions in a randomized order, including standardized lunch at 12:00 (12S); apple preload 30 min prior to 12S (12A+S); standardized lunch at 14:00 (14S); apple preload 30 min prior to 14S (14A+S).

Each participant attended four separate experimental conditions in an unblinded, randomized, crossover design, with each session being separated by a wash-out period of at least three days. As shown in Figure 1, each test session spanned four consecutive days from around 18:00 on Day −1 until 8:00 on Day 2. Participants were advised to maintain a regular sleep routine and not to perform any strenuous exercise during each study session.

The procedures were identical during each test session, except for the type and timing of the served lunch meals on the test day (Day 1). On Day −1, participants were fitted with a continuous glucose monitor (CGM) (Abbott, Shanghai, China) and a smart bracelet (Xiaomi, Beijing, China) to monitor the physical activity during each test session. On Day 0, participants were instructed to consume the breakfast, lunch and dinner in a ‘free-living’ environment at 8:00, 12:00, and 18:00, respectively, and abstain from fruits, coffee, tea or alcohol. On Day 1 (trial day), participants arrived at the laboratory around 7:45 following an overnight fast lasting 12 h and were provided with a standardized breakfast at 8:00. Then, participants were not allowed to consume anything other than water until the lunch meal, which was one of the four test meals: (1) standardized lunch at 12:00 (12S); (2) apple preload 30 min prior to 12S (12A+S); (3) standardized lunch at 14:00 (14S); and (4) apple preload 30 min prior to 14S (14A+S). In the non-preload meal, water was given 30 min before the meal instead of apple. At 18:00, the participants were provided with the traditional Chinese food—dumplings—as the subsequent standardized dinner. The contents and energy of breakfast and lunch were identical for each participant. For the dinner, the participants consumed the dumplings ad libitum during the first trial, and the quantity ingested was replicated in the subsequent trials for each participant.

### 2.3. Test Meal Components

The standardized breakfast containing 383 kcal had a macronutrient composition of approximately 58% carbohydrate, 21% fat and 21% protein, consisting of a sandwich (toast, sliced ham, tomato, lettuce) and a cup of soy milk. The macronutrient contents and energy composition of the test lunch meals are shown in Table 1. The standardized lunch (S) consisted of white rice, lactose-free low-fat milk, egg and a vegetable salad (romaine lettuce, cherry tomato, broccoli with roasted sesame dressing and sesame oil), and 119.2 g water for weight balance. The standardized lunch with apple preload (A+S) included red fuji apple containing 15.0 g of available carbohydrate consumed 30 min prior to a standardized lunch with reduced amount of rice. The two test lunch meals were tightly matched in energy content and macronutrient distribution as 60% from carbohydrate, 25% from fat and 15% from protein. The energy of dumplings for a high-fat dinner were distributed as follows: 54% from fat, 33% from carbohydrate and 13% from protein, with 37 kcal per dumpling. The energy content and macronutrient composition of the served food items were calculated from China Food Composition Tables, manufacturer data and determination experiments. All the test meals were prepared and weighed by the study staff on the day of each session, immediately served to the volunteers and consumed within 15 min to avoid possible retrogradation of starch.

### 2.4. Continuous Glucose Monitoring

The continuous glucose monitor (CGM) was performed on Day −1 at approximately 18:00 and the sensor was removed on Day 2 of the study at approximately 9:00. The data reported in this paper represented interstitial glucose readings recorded every 15 min and occasional missing values were imputed by averaging adjacent values.

### 2.5. Blood Collection and Analysis

During lunch meals, blood glucose concentrations from finger prick samples were measured by a handheld, commercial glucometer (LifeScan Inc., Milpitas, CA, USA) at −40 and −30 min, after which the participants consumed water or apple, and further blood was drawn at −15, 0, 15, 30, 45, 60 and 90 min. In addition, a 150 μL capillary blood sample from the fingertip was collected into EDTA K2-treated centrifuge tubes (WanDGL Ltd., Jinan, China) at −30, 0, 30, 45, 60 and 90 min for insulin measurement. Within 30 min of blood collection the blood samples were centrifuged at 1000×g for 15 min with 60 µL supernatant plasma dispensed into 0.5 mL Eppendorf tubes and stored at −80 °C until the assay. Plasma insulin concentrations were determined using an ELISA-based test kit (JunLB Ltd., Beijing, China).

### 2.6. Data Processing and Statistical Analysis

The postprandial glycemic data analysis was based on the change values relative to the fasting concentration. The postprandial insulin data were based on the percent change in insulin relative to the fasting insulin concentration to eliminate inter-personal variability. The incremental area under the curve (iAUC) was calculated for postprandial interstitial/capillary glucose and insulin responses using the trapezoid method above the baseline concentration. The incremental peak values (∆Peak) for postprandial glucose and insulin were calculated. To assess the postprandial glucose variability, the following indexes were assessed: the largest amplitudes of glucose excursion (LAGE); the standard deviation of blood glucose (SD); the coefficient of variation in blood glucose (CV); continuous overlapping net glycemic action (CONGA-1), defined as the SD of the glycemic changes recorded between a specific point and a point one hour earlier; and J-index, calculated as 0.324 × (mean glucose + SD glucose)^2^. To estimate insulin sensitivity, the insulin sensitivity index was calculated as 10,000/square root of (fasting glucose × fasting insulin × mean glucose × mean insulin) [31]. An index of postprandial insulin resistance (HOMA-PP) was calculated for each lunch trial using the following equation [32]: HOMA-PP (×10^3^) = iAUC glucose × iAUC insulin/22.5, which has been validated against the minimal model and the intravenous glucose tolerance test [33,34].

The analysis of 24 h glycemic response monitored by CGM, covering from 8:00 on Day 1 to 8:00 on Day 2, was based on the absolute glucose concentrations. Total area under the curve (tAUC), the mean glucose concentrations (Mean), the max glucose concentrations (Peak), LAGE and SD were calculated for 24 h glycemic response. Additionally, the percentage of the glucose changes greater than 2.5 for each participant (GC > 2.5) was calculated to reflect the 24 h glucose excursion, as well as the percentage of GC > 5.0. We also defined ∆P_L-D_ as the difference between the postprandial glucose peak values after lunch and dinner to represent the glucose fluctuation over a day.

A power calculation was conducted with the PASS 13 *Power Analysis and Sample Size* software (NCSS, Kaysville, UT, USA), based on a previous study [27]. A sample size of *n* = 11 was required to provide 80% power to detect a change of 167.8 mmol·min/L in iAUC (*p* < 0.05), assuming that the standard deviation (SD) is lower than 55.15 mmol·min/L.

All the statistical analysis was performed using the SPSS version 23.0 (SPSS Inc. Chicago, IL, USA). Two-way repeated measures ANOVA was chosen to assess the effects of treatment, time, and the interaction of treatment and time. One-way analysis of variance ANOVA and Duncan’s multiple range test were performed to analyze the differences in the above-mentioned parameters. The variables are presented as the mean ± standard deviation (SD) or the mean value with standard error (SE), with *p* < 0.05 considered statistically significant.

## 3. Results

### 3.1. Baseline Characteristics of Participants

A total of 26 participants were enrolled in the study and completed four treatments with CGM, wherein 20 participants’ capillary blood was collected for glucose and insulin analysis, as the remaining 6 participants had difficulty in collecting 150 μL capillary blood from the fingertip. Baseline characteristics of study participants are presented in Table 2.

The total daily energy intake on Day 1 was 1646 ± 137 kcal, with a distribution spread of 23% at breakfast, 39% at lunch and 38% at dinner meal. None of the participants reported any physical or gastrointestinal discomfort in each trial.

### 3.2. Postprandial Interstitial Glycemic Responses Following the Lunch Test Meals

**Figure 2:**
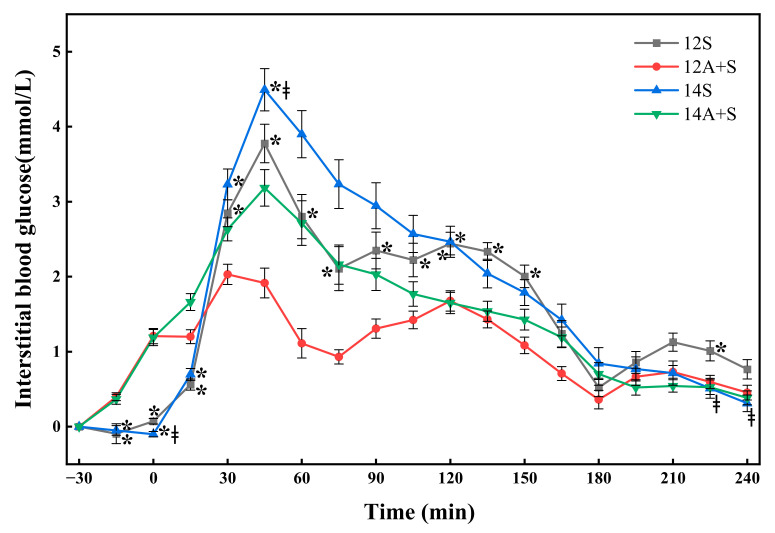
Postprandial interstitial glycemic responses following the lunch test meals. 12S, standardized lunch at 12:00; 12A+S, apple preload 30 min prior to 12S; 14S, standardized lunch at 14:00; 14A+S, apple preload 30 min prior to 14S. −30 min = the commencement of the water or apple; 0 min = the time when the standardized mixed meal was given. Data are mean ± SE (*n* = 26). * Apple preload treatments (A+S) different from their standardized lunch (S) counterparts (*p* < 0.05), ^‡^ 14S different from 12S (*p* < 0.05).

The postprandial interstitial glycemic responses following the lunch test meals are shown in Figure 2. The postprandial interstitial glycemic responses of the 12A+S were remarkably lower than that of 12S, manifesting a significant lower glucose level from 30 min to 150 min and at 225 min. The 14A+S led to a significant lower glucose level at 30, 45 and 120 min than that of 14S. With respect to the different lunch timings, the 14S elicited a lower fasting glucose at 0 min, a higher peak value at 45 min, and lower glucose values at 225 and 240 min than the 12S did (*p* < 0.05). The postprandial glucose response pattern of the early lunch at 12:00 was characterized by three small peaks instead of one sharp peak in the case of the late lunch at 14:00 during the whole 270 min.

Table 3 shows the postprandial interstitial glycemic parameters for the lunch test meals. The apple preload treatments (A+S) elicited significantly lower ∆Peak_270_, LAGE_270_ and other glycemic variability indices than their standardized lunch (S) counterparts, regardless of the meal timing. In addition, the degree of improvement in 12A+S trial conditions was superior to 14A+S for all the parameters. The iAUC_0-270_ of 12A+S was significantly improved and achieved a 33.7% reduction compared with that of the 12S. There were no differences in the postprandial interstitial glycemic parameters except for the ∆Peak_270_ between 12S and 14S. In addition, the numbers of participants who had peak blood glucose concentrations exceeding 10 mmol/L were eight in the 14S test while only two in the 12S test.

### 3.3. Postprandial Capillary Glucose and Insulin Responses Following the Lunch Test Meals

**Figure 3:**
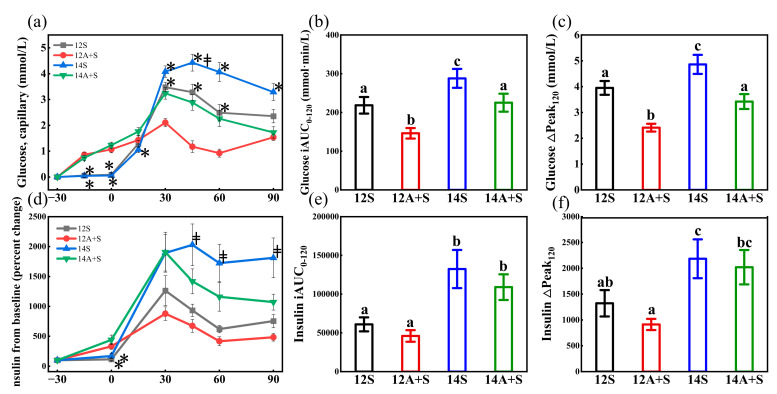
Postprandial capillary glucose responses and parameters (**a**–**c**), postprandial capillary insulin responses and parameters (**d**–**f**) in four lunch trial conditions. 12S, standardized lunch at 12:00; 12A+S, apple preload 30 min prior to 12S; 14S, standardized lunch at 14:00; 14A+S, apple preload 30 min prior to 14S. −30 min = the commencement of the water or apple; 0 min = the time when the standardized mixed meal was given. Data are mean ± SE (*n* = 20). * Apple preload treatments (A+S) different from their standardized lunch (S) counterparts (*p* < 0.05), ^‡^ 14S different from 12S (*p* < 0.05). Significant differences (*p* < 0.05) are represented by different letters on the bars in figure (**b**,**c**,**e**,**f**).

Figure 3 shows the postprandial capillary glucose and insulin responses and parameters in four lunch trial conditions. The outcomes of capillary glucose were generally consistent with those obtained by CGM, except that a delayed peak value occurred at 45 min in the postprandial glycemic response curve and a larger glucose iAUC_0-120_ in 14S (*p* < 0.05). The insulin data showed that the 14S induced higher insulin iAUC_0-120_ and insulin ∆Peak_120_ than 12S did (*p* < 0.05). Apple preload restored the peak glucose concentration and the glucose iAUC after the late lunch but failed to reverse the insulin iAUC to that of the early lunch’s level.

**Figure 4:**
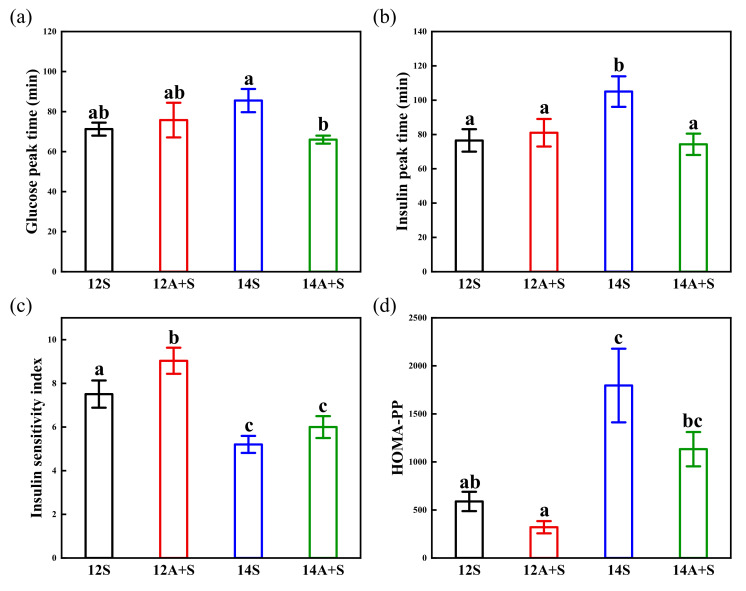
Glucose peak time (**a**), insulin peak time (**b**), insulin sensitivity index (**c**) and HOMA-PP (**d**) in four lunch test meals. 12S, standardized lunch at 12:00; 12A+S, apple preload 30 min prior to 12S; 14S, standardized lunch at 14:00; 14A+S, apple preload 30 min prior to 14S. Data are mean ± SE (*n* = 20). Significant differences (*p* < 0.05) are represented by different letters on the bars.

As shown in Figure 4, there was a significant difference between the 14S and the 14A+S in terms of the glucose peak time. Furthermore, an obvious delay in the insulin peak time was observed in the 14S. The insulin sensitivity index of the 14S was 30.7% lower than that of the 12S, while the 12A+S achieved a 20% increase in the insulin sensitivity index compared with 12S (*p* < 0.05). In addition, the HOMA-PP of 14S was 3.05-fold higher than that of the 12S (*p* < 0.05). The delayed insulin peak time of the 14S was restored, but the impaired insulin sensitivity and insulin resistance was not recovered by the apple preload treatment (14A+S).

### 3.4. Postprandial Interstitial Glycemic Responses Following the Subsequent Meals

**Figure 5:**
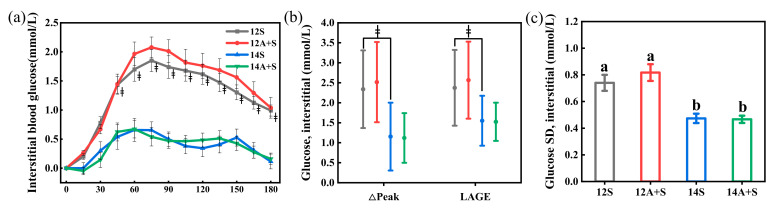
The postprandial interstitial glycemic response (**a**), the ∆Peak and LAGE (**b**), and the SD (**c**) at a subsequent high-fat dinner. 12S, standardized lunch at 12:00; 12A+S, apple preload 30 min prior to 12S; 14S, standardized lunch at 14:00; 14A+S, apple preload 30 min prior to 14S. 0 min = the commencement of the dinner. Data are mean ± SD in figure (**b**), mean ± SE in other figures. ^‡^ 14S different from 12S (*p* < 0.05). Significant differences (*p* < 0.05) are represented by different letters on the bars in figure (**c**).

Figure 5 shows the postprandial interstitial glycemic response and parameters after a high-fat dinner, the subsequent meal of the test lunch. There were no differences in the postprandial interstitial glycemic responses and parameters between A+S and S, while the meal timing made a difference indicated by a higher glucose value from 45 to 180 min and increased ∆Peak, LAGE, SD in the subsequent meal of 12S (*p* < 0.05). The difference in the postprandial glucose iAUC_0-180_ after dinner between S and its A+S counterparts did not reach the significant level, though about half of the subjects showed increased glycemic iAUC_0-180_ after the dinner following the apple preload lunch meals. 

### 3.5. 24 h Interstitial Glucose Trace

**Figure 6:**
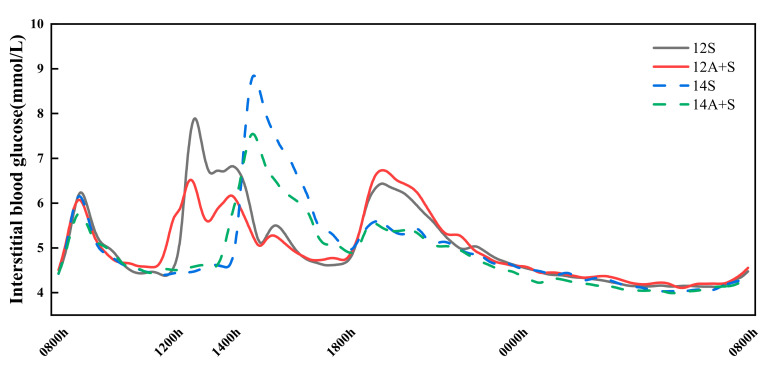
The interstitial glucose trace for 24 h in lunch testing trial conditions. 12S, standardized lunch at 12:00; 12A+S, apple preload 30 min prior to 12S; 14S, standardized lunch at 14:00; 14A+S, apple preload 30 min prior to 14S.

Figure 6 shows the interstitial glucose trace for 24 h in four lunch test trials, and the 24 h interstitial glucose parameters are shown in Table 4. There was no significant difference in the 24 h Mean and 24 h tAUC among the four lunch trial conditions. The apple preload intervention (A+S) elicited significantly lower 24 h Peak, LAGE, SD and ∆P_L-D_ than their non-preload counterparts did, regardless of the lunch meal timing. The treatment 12A+S attained a smallest percentage of the glucose changes greater than 5.0 mmol/L (GC > 5.0), while the 14A+S achieved a significant decrease in the percentage of GC > 2.5 and GC > 5.0 compared with the 14S. The 24 h Peak, LAGE, the percentage of GC > 5.0 and ∆P_L-D_ of the 14S were significantly higher than those of the 12S.

## 4. Discussion

The present study found that the postprandial glycemic response following a late lunch (14S), which had an extra 2-h interval between the breakfast and the lunch, was characterized by significantly elevated glucose peak, delayed insulin peak time, decreased insulin sensitivity index, increased insulin resistance following the lunch, and also decreased glycemic response following the subsequent dinner and larger blood glucose fluctuation over the 24-h period when compared with an early lunch (12S). The apple preload prior to a mixed meal with balanced macronutrient composition could partly reverse the negative impact of a late lunch by lowering the glucose peak, restoring the insulin peak time, reducing the glycemic variability, and improving the 24-h glycemic variability. The apple preload treatment improved the insulin sensitivity when applied at an early lunch, but failed to recover the insulin sensitivity at a late lunch.

Several crossover trials have shown the detrimental glucose metabolism consequences caused by late dinner in healthy participants [12,13,14,35,36], but only one study investigated the effect of a late lunch on postprandial blood glucose levels [23]. In the present study, the early and late lunch were set at 12:00 and 14:00, respectively, with a 2-h difference between meals, while the previous study was set at 13:00 and 16:30, respectively, with a 3.5-h difference between the two treatments [23]. Though there was only a 2-h difference in breakfast–lunch intervals, we observed a significant higher peak glucose and insulin value, decreased insulin sensitivity index, and greater postprandial insulin resistance in the late lunch trial condition (14S) compared with that of the early lunch.

The early-phase insulin response was supposed to play a critical role in determining postprandial hyperglycemia [37,38]. In the present study, compared with the 12S, the 14S showed delayed insulin peak synchronized with a delayed and elevated glucose peak, suggesting that the late lunch might induce a weak early-phase insulin secretion due to an insufficient β-cell response, which in turn posed a need for a sustained insulin supply to bring the glucose concentration back towards the baseline.

The larger postprandial glucose fluctuation in the 14S could be attributed to the prolonged meal interval between breakfast and lunch resulting from late eating, which might change the energy substrate utilization as fasting carbohydrate oxidation would decrease under a late eating condition [23]. Previous studies observed that breakfast skipping increased fat oxidation and decreased carbohydrate oxidation before lunch [39,40], while the increased preprandial lipid oxidation could be an independent predictor of postprandial glycemic response [41]. A prolonged fasting period might be considered as a stress that leads to enhanced lipolysis in both healthy subjects [42] and the type 2 diabetes [43]. An elevated plasma free fatty acid level, however, was associated with increased insulin resistance and hepatic glucose production [44,45].

The circadian rhythm could be another possible factor in the postprandial hyperglycemia in the condition of a late lunch, as the endogenous rhythms promote a steady increase in glucose concentration through the 24-h day independent of the behavioral cycle [46,47]. The misalignment between the circadian cycle and the fasting/feeding and sleep/wake cycle has been shown to elevate both the glucose and insulin level due to the exaggerated postprandial glucose response in the later part of day [48]. However, in the late-night feeding trials, the intervals between the lunch and dinner were different (5 h vs. 9 h [12], 5 h vs. 8 h [13], 6 h vs. 9 h (with a snack in the afternoon) [14], 7 h vs. 10.5 h [35] and 6 h vs. 11 h [36]) in the previous studies. The interval between meals might play a larger role in effecting the postprandial glycemic response, in addition to the circadian factor. The outcome of the present study supported our research hypothesis, as a 2-h longer interval between breakfast and lunch resulted in a poorer glycemic response after lunch, while a 2-h shorter lunch–dinner interval induced a milder glycemic response after dinner.

The window for food consumption in this study was 10 h (between 8:00 and 18:00). It is reported that a mild time-restricted eating (TRE), especially those focused on the early part of the day [49], could improve blood glucose control and insulin sensitivity [14,15]. The present study suggests a possibility that part of the benefit of the TRE might be attributed to the shortened meal interval in addition to the circadian rhythm factor, since the timing of the intermediate meal influenced the 24-h glucose fluctuation, with a fixed eating schedule of first and last meal. Given that most studies on TRE only specified the eating window between the first and last meal [50,51], the effect of the interval between meals deserves investigation in future studies.

Previous studies have confirmed the hypoglycemic effect of the apple preload based on a rice meal [27,29]. However, this study is the first to show that the apple preload could ameliorate the postprandial glycemic response following a mixed meal, whether at a routine lunch time or at a late lunch setting, based on a nutrient-balanced meal composition. The apple preload effectively reversed some of the negative effects of a later lunch, achieved a smaller blood glucose peak, a non-delayed glucose peak time and insulin peak time, reduced iAUC of blood glucose, and milder glycemic excursion compared with its non-preload counterpart.

The insulin data indicated that the apple preload did not elicit increased absolute insulin secretion. On the contrary, it was found that the insulin iAUC_0-120_ of 12A+S and 14A+S achieved a 24.5% and 17.6% decrease, respectively, compared with their non-preload counterparts. The insulin levels were lower in 12A+S at 60–120 min and 14A+S at 75–120 min, compared with their non-preload counterparts. What is more, the 12A+S showed improved postprandial insulin sensitivity index compared with the 12S. These results suggested that the hypoglycemic effect of the apple preload might relate to an enhanced insulin sensitivity rather than elevated insulin production.

The mechanism of the apple preload’s action on the improvement of postprandial glycemic response is yet to be elucidated. As our previous study found that the co-ingestion of apple and rice did not elicit the same effect as apple preload 30 min prior to the rice did [27], it seems that the effect of the preload treatment depends on a precise timing [52]. The sugar component of apple may play an important role, as the fructose [53] and apple sugar solution [27] could attenuate the postprandial glycemic response in a preload setting. Though the fructose does not stimulate a rapid insulin response, the ingestion of sugars such as glucose and sucrose can be a stimulus for the cephalic phase insulin response (CPIR) that helps to improve glucose control [54,55]. The action of CPIR could not be ruled out though the insulin data within 10 min was not collected. It is possible that a synergy of the induction of CPIR and the catalytic effect of fructose on liver glucose metabolism [56,57] enabled the remarkable preload effect without up-regulating the insulin, as a greater fructose/glucose ratio in the moiety of sugar components was found to favor the hypoglycemic effect of fruits and dried fruits [58]. Another possibility is the modification of the pattern of entero-insular axis peptides by preload apple carbohydrate. It is reported that slow-digest carbohydrates could increase glucagon-like peptide-1(GLP-1) while decreasing gastric inhibitory polypeptide (GIP) [59]. The carbohydrate in apple is of low glycemic type with glycemic index of 36 [60], while apple polyphenols were shown to be associated with the inhibition capacity of α-glucosidase [61].

To the best of our knowledge, this is the first study to explore the effect of between-meal interval on postprandial glycemic effect of two meal settings with two macronutrient composition meals. It is also the first report with respect to the effect of a preload on the hyperglycemia caused by delayed meals. The glucose and insulin responses during the postprandial period based on different lunch timing were investigated, as well as the glycemic response following the next meal. The glycemic effect of the test meals was double checked with CGM and capillary blood tests. In this study, the mealtime on Day 0 was stipulated and the breakfast on Day 1 was provided for the participants in order to minimize any variations in baseline metabolite concentrations prior to the lunch trial and diminish possible carryover effects derived from the participant’s previous meal. Given that the irregular mealtime is a common problem in working people of contemporary society, the study on the glycemic response based on meal intervals is relevant.

However, the limitations of our study must be considered. Firstly, this is a one-day experiment with healthy participants, so the effect of delayed lunch and prolonged inter-meal fasting is still to be confirmed by long-term intervention studies and in the prediabetic and the diabetic groups. Secondly, the CPIR after meal was not determined. Thirdly, the possible change in the level of hormones such as cortisol and GLP-1, as well as the possible change in digestion process, were not investigated. Finally, only the apple preload was tested in the trial. The possible preload effect of other food could not be extrapolated from the present study.

## 5. Conclusions

In conclusion, the present study found that an extra 2-h inter-meal fasting before and after lunch could result in elevated glycemic response in both macronutrient-balanced meals and high-fat meals in healthy participants. The negative impact of a late lunch could be partly reversed by an apple load prior to lunch, without a trade-off of insulin secretion. The result of the present study suggests that the between-meal interval may be a potential key determinant in glycemic stability in addition to the diurnal rhythm. The possible metabolic consequences of late lunch times and the mechanism need to be investigated, especially in people of impaired glucose tolerance and the diabetes patients.

## Figures and Tables

**Table 1:** The composition, macronutrient and energy contents of the lunch test meals ^1^.

Test Meals	Carbohydrate (g)	Protein (g)	Fat (g)	Energy (kcal)	Detail Content
S ^2^	97.2	24.2	18.0	648	Lactose-free low-fat milk 200 g, roasted sesame dressing 25 mL, egg yolk 10 g, egg white 50 g, romaine Lettuce 25 g, cherry tomato 75 g, broccoli 50 g, sesame oil 2 g, uncooked rice 100 g, water119.2 g
A+S ^3^	97.2	24.1	18.1	648	Lactose-free low-fat milk 200 g, roasted sesame dressing 25 mL, egg yolk 10 g, egg white 50 g, romaine Lettuce 25 g, cherry tomato 75 g, broccoli 50 g, sesame oil 1 g, uncooked rice 80.2 g, apple 140 g

^1^ The nutritional contents of the lunch test meals were obtained from China Food Composition Tables, manufacturers and determination experiments.^2^ S: standardized lunch.^3^ A+S: apple preload 30 min prior to standardized lunch.

**Table 2:** Baseline characteristics of study participants.

Characteristics	Mean ± SD (Male/Female)
Number of participants (male/female)	26(12/14)
Age, years	20.8 ± 0.9
Body composition	
BMI, kg/m^2^	21.4 ± 2.1/20.8 ± 1.7
Waist: hip ratio	0.7 ± 0.0/0.8 ± 0.0
Waist: height ratio	0.4 ± 0.0/0.4 ± 0.0
Fat mass, %	15.6 ± 4.1/24.1 ± 4.0
Visceral fat index	4.8 ± 2.0/2.3 ± 1.2
Basal metabolic rate (BMR), kcal/day	1391.6 ± 203.4
Systolic blood pressure, mmHg	114.0 ± 12.3
Diastolic blood pressure, mmHg	67.0 ± 9.0
Habitual meal timing	
Breakfast	8:02 ± 0:42
Lunch	11:42 ± 0:25
Dinner	17:41 ± 0:22

**Table 3:** Postprandial interstitial glycemic parameters for the lunch test meals (mean ± SE, *n* = 26).

Test Meals	iAUC_0-270_ (mmol·min/L)	∆Peak_270_ (mmol/L)	LAGE_270_ (mmol/L)	SD	CV (%)	CONGA-1	J-Index
12S	432.6 ± 29.7 ^a^	3.9 ± 0.2 ^a^	4.2 ± 0.3 ^ab^	1.3 ± 0.1 ^ab^	21.1 ± 1.1 ^ab^	2.0 ± 0.1 ^ab^	17.1 ± 0.8 ^ab^
12A+S	287.0 ± 18.3 ^b^	2.4 ± 0.1 ^b^	2.5 ± 0.1 ^c^	0.7 ± 0.0 ^c^	12.7 ± 0.6 ^c^	1.1 ± 0.1 ^c^	13.0 ± 0.5 ^c^
14S	482.1 ± 34.6 ^a^	4.7 ± 0.3 ^c^	5.0 ± 0.2 ^a^	1.6 ± 0.1 ^a^	24.6 ± 1.1 ^a^	2.5 ± 0.1 ^a^	20.5 ± 1.2 ^a^
14A+S	392.0 ± 26.9 ^a^	3.4 ± 0.2 ^a^	3.5 ± 0.2 ^b^	1.0 ± 0.1 ^b^	17.4 ± 1.1 ^b^	1.6 ± 0.1 ^b^	15.9 ± 0.8 ^b^

^a,b,c^ Different superscript letters denote that mean values within a column are significantly different (*p* < 0.05). 12S, standardized lunch at 12:00; 12A+S, apple preload 30 min prior to 12S; 14S, standardized lunch at 14:00; 14A+S, apple preload 30 min prior to 14S.

**Table 4:** The 24 h interstitial glucose parameters for four lunch trail conditions (mean ± SE, *n* = 26).

Test Meals	24 h Mean (mmol/L)	24 h tAUC (mmol·h/L)	24 h Peak (mmol/L)	24 h LAGE (mmol/L)	24 h SD	GC > 2.5 (%)	GC > 5.0 (%)	∆P_L-D_
12S	5.0 ± 0.1	120.8 ± 1.5	8.4 ± 0.2 ^a^	4.7 ± 0.3 ^a^	1.0 ± 0.0 ^ac^	6.1 ± 0.8 ^a^	0.2 ± 0.1 ^a^	1.4 ± 0.2 ^a^
12A+S	5.0 ± 0.1	120.2 ± 1.8	7.4 ± 0.2 ^b^	3.6 ± 0.2 ^b^	0.9 ± 0.0 ^b^	4.0 ± 1.0 ^ab^	0.0 ± 0.0 ^a^	0.3 ± 0.2 ^b^
14S	5.0 ± 0.1	119.5 ± 1.7	9.4 ± 0.3 ^c^	5.7 ± 0.3 ^c^	1.1 ± 0.1 ^c^	5.9 ± 0.7 ^a^	1.3 ± 0.4 ^b^	3.3 ± 0.2 ^c^
14A+S	4.9 ± 0.1	116.6 ± 2.0	8.0 ± 0.2 ^ab^	4.1 ± 0.2 ^ab^	0.9 ± 0.0 ^ab^	3.6 ± 0.6 ^b^	0.2 ± 0.1 ^a^	2.1 ± 0.3 ^d^

^a,b,c,d^ Different superscript letters denote that mean values within a column are significantly different (*p* < 0.05). 12S, standardized lunch at 12:00; 12A+S, apple preload 30 min prior to 12S; 14S, standardized lunch at 14:00; 14A+S, apple preload 30 min prior to 14S.

## Data Availability

The data presented in this study are available on request from the corresponding author.
